# Concurrent Validity and Between-System Agreement of a Commercial Wearable Inertial Sensor System for Gait and Postural Sway Assessment in Progressive Supranuclear Palsy

**DOI:** 10.3390/s26165105

**Published:** 2026-08-12

**Authors:** Ryan E. Novotny, Victor S. You, Cecilia A. Hogen, Jennifer L. Whitwell, Keith A. Josephs, Kenton R. Kaufman, Farwa Ali

**Affiliations:** 1 Neurology, Mayo Clinic, Rochester, MN 55905, USA; novotny.ryan@mayo.edu (R.E.N.); you.sungmin@mayo.edu (V.S.Y.); josephs.keith@mayo.edu (K.A.J.); 2 Motion Analysis Laboratory, Mayo Clinic, Rochester, MN 55905, USA; hogen.cecilia@mayo.edu (C.A.H.); kaufman.kenton@mayo.edu (K.R.K.); 3 Radiology, Mayo Clinic, Rochester, MN 55905, USA; whitwell.jennifer@mayo.edu

**Keywords:** Progressive Supranuclear Palsy, inertial measurement units, wearable sensors, gait analysis, postural sway, kinematics, atypical parkinsonism

## Abstract

Wearable inertial measurement units (IMUs) offer an accessible alternative to optical motion capture (MoCap) gait analysis, but their performance in Progressive Supranuclear Palsy (PSP) requires validation. We assessed the concurrent validity of IMU-derived versus MoCap-derived gait metrics and static postural sway in 30 patients with PSP using Bland–Altman analysis, Intraclass Correlation Coefficients (ICC), and Spearman rank correlations. Finally, we assessed equivalence using the Two one-sided tests (TOST) procedure. Multivariable linear regression was used to determine whether clinical severity, as measured by the PSP Rating Scale (PSPRS), independently predicted absolute IMU measurement error while controlling for patient age and gait velocity. IMUs demonstrated excellent between-system agreement for parameters such as cadence (100.76 ± 11.42 vs. 100.52 ± 11.59) and cycle time (1.21 ± 0.15 vs. 1.22 ± 0.15; ICC > 0.98), despite a systematic underestimation of gait velocity (*p* < 0.05). Agreement significantly diminished for micro-phases (e.g., single/double support times) and spatial asymmetry. Interestingly, the TOST procedure revealed that only sagittal and transverse trunk kinematics were equivalent between systems, with all other measures failing to find equivalency. For static sway, the IMU demonstrated strong rank-order correspondence for tracking relative postural instability (ρ = 0.82, *p* < 0.05). Multivariable analysis revealed that higher PSPRS scores are independently associated with greater between-system discrepancies in support phases and pelvic and trunk kinematics (*p* < 0.05), irrespective of reduced gait speed. These findings highlight the need to develop disease-specific algorithms, rather than relying on normative commercial models, to establish reliable digital biomarkers for monitoring progressive motor decline.

## 1. Introduction

Stable gait mechanics and adequate spino-pelvic range of motion (ROM) are essential for independent living and for the safe performance of activities of daily living. In neurodegenerative disorders, restricted mobility and postural instability significantly contribute to the overall burden of disease, and exponentially increasing the risk of fatal falls [1,2]. Progressive Supranuclear Palsy (PSP) is a rapidly progressive, atypical parkinsonism characterized clinically by vertical supranuclear gaze palsy, axial rigidity, early and severe postural instability, and abnormal gait mechanics [3]. Specifically, patients with PSP frequently exhibit a backward-leaning posture [4], restricted trunk kinematics [5], and severe postural instability and imbalance [3,6], which impairs mobility and drives a high frequency of unexplained falls [7]. Therefore, accurate quantification of gait and kinematic abnormalities is critical for monitoring disease progression and evaluating the efficacy of interventions in clinical trials.

Traditionally, optical motion capture (MoCap) systems have been the gold standard for assessing gait and ROM [8]. However, MoCap is generally confined to specialized biomechanical laboratories due to need for space and highly trained personnel, limiting its utility for routine clinical monitoring. Conversely, wearable inertial measurement units (IMUs) present as a clinically accessible alternative for detecting changes in kinematics and spatiotemporal variables [9]. Additionally, IMU devices can potentially be used outside lab and clinic settings, enabling remote monitoring at home and throughout daily life [10].

Despite increasing in popularity, the clinical application of out-of-the-box commercial IMU software relies on proprietary algorithms derived from normative biomechanical models. While other research grade sensors provide unprocessed data that researchers may then analyze with custom, open-source algorithms or scripts, these approaches require bioengineering expertise which may not be available in clinical settings. Consequently, clinics will likely rely on commercial systems that automatically generate discrete metrics. In a prior study, we evaluated the accuracy of a commercial 6-sensor APDM Opal IMU system (APDM Wearable Technologies, Portland, OR, USA) and its accompanying software in a cohort of healthy adults, demonstrating valid and reliable trunk and pelvis ROM measurements compared with a traditional MoCap system [11]. In healthy young and older adults, the system has demonstrated high concurrent validity for foundational gait metrics when compared to instrumented treadmills and instrumented walkways [12]. The system has also proven useful in quantifying mobility deficits in distinct neurological populations, with validation studies showing that the system accurately captures gait abnormalities in Parkinson’s disease [13] and can identify patterns of gait variability in patients with cerebellar ataxia [9]. However, it remains unknown whether the accuracy and reliability of this commercially available IMU system algorithm hold when applied to severe, pathological gait observed in individuals with PSP. Since PSP is a rare disease that may be misdiagnosed as Parkinson’s Disease [14] where gait and balance abnormalities are early and profound [15], there is limited literature detailing how different movement analysis modalities compare [10]. Investigating device performance within this population therefore represents a critical step toward establishing reliable digital biomarkers.

Therefore, the primary objective of this study was to evaluate the concurrent validity and absolute agreement of trunk and pelvic ROM, alongside spatiotemporal gait metrics, measured using the commercially available APDM Opal IMU system compared to a gold-standard marker based MoCap system in a cohort of patients diagnosed with PSP. A secondary objective was to use multivariable linear regression to determine whether clinical disease severity, as measured by the PSP Rating Scale (PSPRS), is independently associated with discrepancies in the accuracy of the IMU sensor’s measurement algorithms.

## 2. Materials and Methods

### 2.1. Participant Recruitment

Participants were recruited from the Neurodegenerative Disease Research Group (NRG). Participants were included if they met the 2017 Movement Disorders Society PSP (MDS-PSP) [3] criteria applied by movement disorders neurologists (FA, KAJ), were not bed/wheel chair bound and were not on any medications that would affect symptoms or gait and balance. Participants underwent neurological exams, PSPRS evaluation, and assignment of PSP subtype label per the MDS-PSP criteria. All participants provided written, informed consent, and the study was approved by the Mayo Clinic Institutional Review Board (IRB# 23-004889, approved 24 May 2023). The experimental protocol and data processing methodologies employed here are identical to those previously published in our prior work [11], and are also described in detail below.

### 2.2. Gait Analysis Method

Participants were instrumented with two simultaneous motion-tracking systems. A six-sensor APDM Opal IMU system recording at 128 Hz was used and each sensor was secured directly to the participants’ skin using double-sided tape at the sternum and sacrum and attached via straps to the bilateral wrists and dorsum of the feet. IMU data collection and kinematic processing were performed by APDM’s commercially available Mobility Lab software (v2.0.2). A 14-camera optical motion capture system (Raptor-12, Motion Analysis Corporation, Santa Rosa, CA, USA) recording at 120 Hz was also utilized as the reference standard. Retroreflective markers for motion capture were placed on the participants following the Helen Hayes configuration [16]. To synchronize the IMU and MoCap systems, a sound-activated circuit was constructed and used as an input to the MoCap system via analog (±2.5 V, 2000 Hz). An audible “Start” and “Stop” cue was used to activate and time-sync the IMU software to the MoCap system and instruct the patient to begin the trial.

Participants were instructed to walk at a comfortable, self-selected pace across a 7 m level walkway, while being secured in a ceiling mounted harness that provided no body weight support and accompanied by a physical therapist to ensure safety and to verify that sensors and markers remained correctly affixed. This procedure was repeated for a minimum of 8 walking trials per participant to ensure adequate data capture. Three-dimensional marker trajectories were captured during the walking trials and were visualized using Cortex Software (v10.0.0.46; Motion Analysis Corporation, Santa Rosa, CA, USA). The data were pre-processed within Cortex and exported to Visual3D (v2023.10.1; C-Motion Inc., Germantown, MD, USA) for post-processing. The segment and pelvic coordinate systems were defined according to the International Society of Biomechanics standards [17].

Walking trials were then assessed for quality (i.e., minimal MoCap marker occlusion, data successfully streamed from all IMU sensors) and ensuring that proper time-synchronization occurred between the IMU and MoCap systems. Each trial consisted of two sequential strides at the center of the walkway, and the three steady-state walking trials best representing comfortable walking without acceleration or deceleration based solely on the MoCap system were then selected for each participant.

### 2.3. Static Sway Assessment

After completing the walking trials, participants’ static postural sway using both systems was evaluated. A force plate setup (AMTI Optima HPS Force Plates, Model OPT400600-HF-1K-CTT; AMTI Signal Conditioners, Model OPT-SC; AMTI, Watertown, MA, USA) recorded sway data at 2000 Hz and was time-synced with the MoCap system. Participants were instructed to stand barefoot on a firm surface with their feet shoulder width apart, each foot on a separate force plate, with their eyes open (EO) and eyes closed (EC). For each task, a single 30 s trial was recorded. Force plate data was subsequently filtered in Visual3D with a fourth order lowpass Butterworth filter with a cut-off frequency of 10 Hz. The center-of-pressure (COP) data was then filtered in MATLAB (R2021b, MathWorks Inc., Natick, MA, USA) with a first order lowpass Butterworth filter with a cutoff frequency of 5 Hz.

### 2.4. Gait Event Detection

The Mobility Lab software utilized a proprietary algorithm to automatically detect gait events and compute discrete measurements of multiple gait parameters. While this algorithm is closed source, it generally utilizes kinematic features from the foot sensors, specifically angular velocity and acceleration, to identify heel-strike and toe-off events, and then integrates these measures to generate spatiotemporal variables [18]. We selected variables measured by both IMU system and Mocap including spatial and temporal parameters, asymmetry, and kinematic ROM of the pelvis and trunk, yielding 17 variables in total for analysis. For the MoCap system, the recorded marker trajectories were filtered within Visual3D with a generalized cross validatory smoothing filter [19] using a smoothing parameter of 0.0001, and a fourth order lowpass Butterworth filter with a cutoff frequency of 6 Hz using MATLAB. Visual3D’s Automatic Gait Detection algorithm was used to characterize gait events, such as initial contact and toe-off, using a combination of force plate data and pattern recognition on foot marker trajectories [20]. After, Visual3D automatically computed spatiotemporal variables based on the detected events. Asymmetry metrics were computed as the percent difference between the right and left sides, with positive values indicating greater difference on the right side. Each trial’s segment angles were then normalized to a gait cycle of 100 frames. Anatomical segment modeling for the trunk and pelvis was performed using Visual3D, calculating the reference ROM as the difference between the maximum and minimum segment angles during the steady-state strides. Variables were then averaged across strides, trials, and sides.

### 2.5. Statistical Analysis Methods

Concurrent validity and agreement between the IMU and MoCap systems were evaluated using Bland–Altman analysis to determine the mean bias and 95% limits of agreement. Wilcoxon Signed Rank tests were used to evaluate significant mean differences between the two systems, and a two one-sided test (TOST) procedure was used to further describe the equivalence of both measurement systems. The TOST requires an equivalency bound to be defined for each variable before running the procedure. While prior studies have shown that a change in velocity of 0.05 m/s is enough to elicit a clinically identifiable difference in Parkinson’s patients and older populations [21,22], there are no established differences specifically for PSP. Due to this, the equivalence bound for remaining values was determined as one half of the MoCap systems standard deviation. Since the bounds utilized here are based on recorded data that may change between cohorts with varying levels of heterogeneity, this analysis is identified as exploratory in nature.

Mean Absolute Error (MAE) and Root Mean Square Error (RMSE) were calculated to quantify absolute measurement error in real-world units. To assess absolute agreement between methods, Intraclass Correlation Coefficients were computed using the *psych* package in R based on a two-way random effects model with absolute agreement for single measurements (ICC(2,1)), testing the null hypothesis that ICC = 0. This model was selected to generalize findings beyond this specific setup, treating the measurement systems as random samples from a broader population of clinical tools. Here, ICC values > 0.90 represent excellent agreement and values < 0.50 represent poor agreement [23]. Furthermore, to determine the effect of pathological gait on sensor accuracy, Spearman Rank Correlations were conducted between the IMU error and clinical severity. Subsequently, multivariable linear regression models were employed to determine if PSPRS independently predicted absolute measurement error while controlling for patient age and gait velocity. We additionally included MoCA scores in another model with patient age and velocity to determine if participants cognition would influence the ability of PSPRS to predict measurement errors.

Since the MoCap system measures sway as a linear center of pressure displacement, while the IMU system measures sway as angular kinematic tilt, concurrent validity for sway variables was assessed using Spearman Rank Correlation. Romberg Ratios were additionally calculated for both systems to quantify sensory reliance. The MoCap system computed the ratio as the amount of sway in center of pressure during the eyes closed task divided by the sway in the eyes open task, and the IMU as the amount of sway in degrees during both tasks. The ratios were then evaluated using the same ICC model and Bland–Altman analysis. Given the exploratory pilot nature of this study (*n* = 30), unadjusted *p*-values are reported alongside effect sizes to balance type I and type II error control. All statistical analyses were performed in RStudio version 2026.04.0+526 with R version 4.6.0, and all significance levels were set to 0.05.

## 3. Results

### 3.1. Cohort Description

A total of 30 (11 female, age 69 ± 8, BMI 28 ± 5) participants diagnosed with PSP were recruited for this study from the NRG at Mayo Clinic Rochester, MN. The cohort comprised individuals with PSP-Richardson’s Syndrome (RS; *n* = 17), subcortical (*n* = 10), and cortical variants (*n* = 3) diagnosed per the 2017 Movement Disorders Society PSP diagnostic criteria [3]. The mean and standard deviation PSPRS and MoCA scores across the cohort were 32 ± 13 and 22 ± 4, respectively, reflecting a spectrum of clinical disease severity. The duration of the diagnosis to exam date across participants was 4.59 ± 2.69 years.

### 3.2. Concurrent Validity and Equivalence

**Figure 1:**
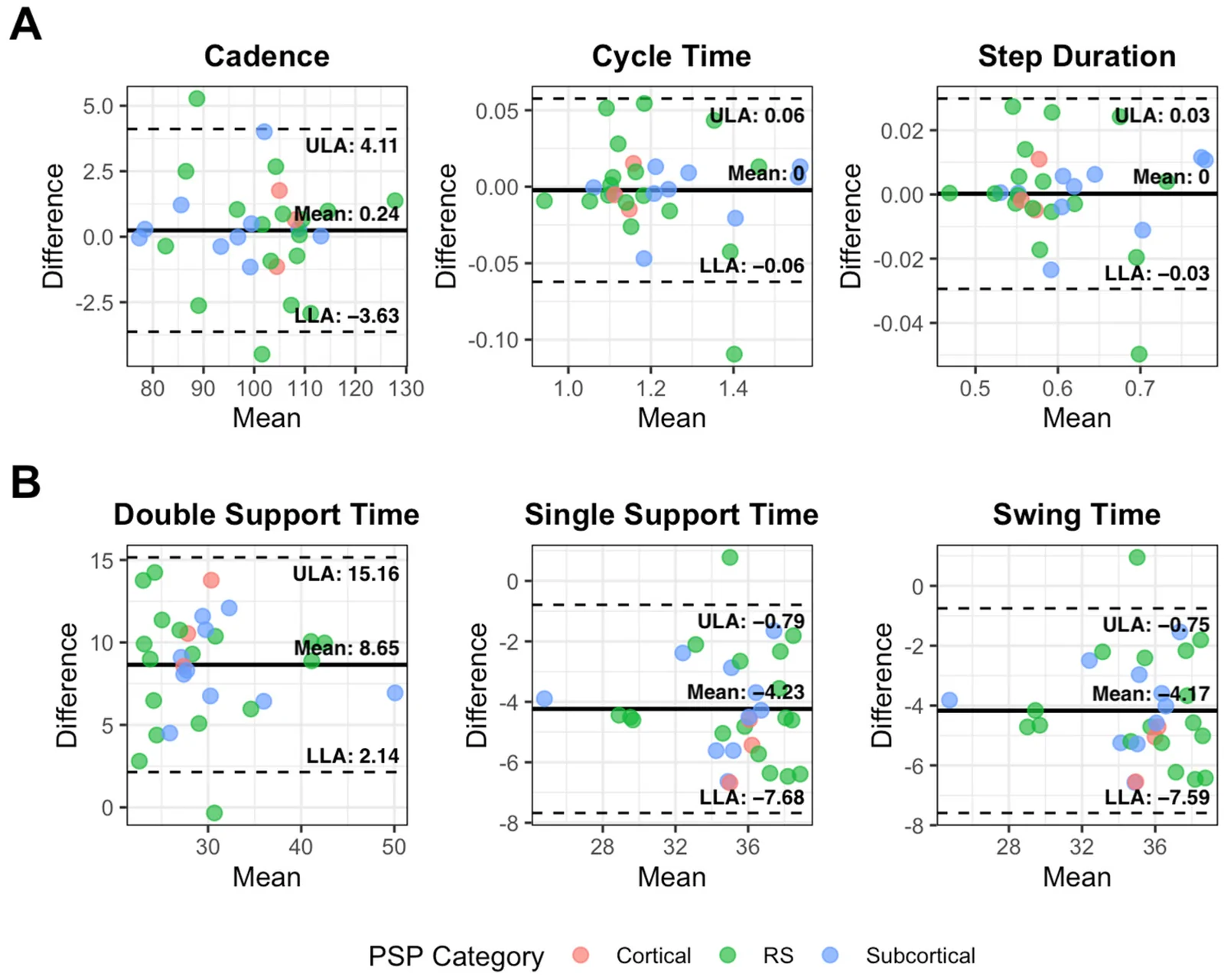
Bland–Altman plots for (**A**) macro- and (**B**) micro-temporal parameters by PSP phenotype. The *x*-axis shows the mean value; the *y*-axis shows the mean differences between MoCap and the IMU.

The IMU demonstrated robust validity for temporal metrics of the gait cycle with Bland–Altman analysis (Figure 1A). No statistically significant differences were observed between the MoCap and IMU means for cadence (100.76 ± 11.42 steps/min vs. 100.52 ± 11.59 steps/min, *p* = 0.382), cycle time (1.21 ± 0.15 s vs. 1.22 ± 0.16 s, *p* = 0.871), or step duration (0.61 ± 0.07 s vs. 0.61 ± 0.08 s, *p* = 0.543). While both systems captured a similar spread across all participants, the IMU underestimated velocity compared to the MoCap system (79.48 ± 19.23 cm/s vs. 71.99 ± 18.63 cm/s, *p* < 0.05), indicating a consistent systematic bias. The TOST procedure revealed that none of the temporal metrics achieved equivalence between the two systems (*p* > 0.05; Table 1), indicating that measurement error exceeded the defined equivalence bounds. Considering that the TOST procedure checks an upper and lower bound in determining equivalence, any measurements that falls outside of the pre-defined bounds will have a negative impact on the variable’s equivalency.

Agreement significantly diminished when analyzing specific sub-phases of the gait cycle (Figure 1B). The IMU generated significant differences in mean double support time (*p* < 0.05), single support time (*p* < 0.05), and swing time (*p* < 0.05). Furthermore, temporal asymmetry showed poor agreement, with the systems failing to align on step duration asymmetry (*p* = 0.289). Notably, the IMU systematically underestimated stride length compared to the MoCap system (84.85 ± 17.16 vs. 94.14 ± 17.58, *p* < 0.05). Since the IMU accurately captured temporal phases, the underestimation of stride length may directly account for the slower gait velocities observed in the temporal results, even though the overall correlations for both metrics remain strong. The TOST procedure showed a complete lack of equivalence for all gait cycle sub-phases, spatial metrics, and asymmetry values (*p* > 0.05).

Validity in kinematic ROM varied significantly by anatomical plane. Trunk ROM in the sagittal and transverse planes showed marginal but statistically significant mean offsets (*p* < 0.05), whereas coronal Trunk ROM showed no significant difference (*p* = 0.529). Pelvic ROM exhibited similar planar dependencies, with the transverse (*p* < 0.05) and sagittal (*p* < 0.05) planes demonstrating the largest mean bias. Here, the TOST procedure identified sagittal and transverse trunk ROM as the only equivalent variables between both systems (*p* < 0.05). Mean Absolute Error (MAE) and Root Mean Square Error (RMSE) for all metrics to capture magnitude of differences are also detailed in Table 2. Expanded metrics for the Bland–Altman and TOST procedures, including confidence intervals and equivalence bounds, may be found within Supplemental Tables S1 and S2.

### 3.3. Between-System Agreement

**Figure 2:**
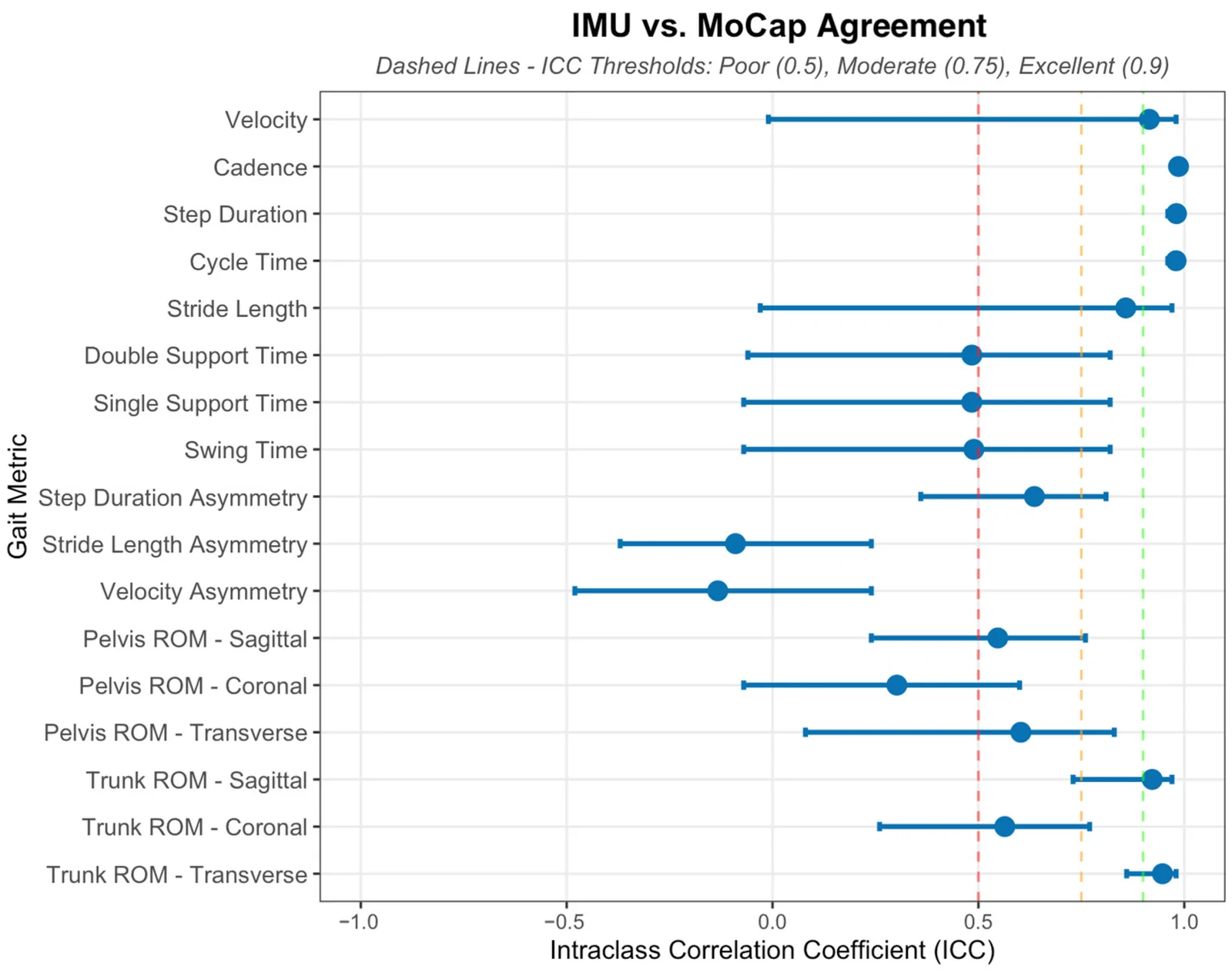
Intraclass Correlation Coefficients for all 17 metrics. Dashed lines indicate standard agreement thresholds (red = poor, yellow = moderate, green = excellent).

Intraclass Correlation Coefficients were calculated to quantify the between-system agreement of the IMU sensors (Figure 2). Global ICC results across the entire cohort mirrored the validity findings. Excellent absolute agreement (ICC > 0.90) was observed for velocity (0.915), cadence (0.986), step duration (0.981), cycle time (0.980), sagittal trunk ROM (0.922), and transverse trunk ROM (0.947). Stride length exhibited moderate-to-good agreement (ICC = 0.858) despite the IMU’s systematic underestimation of the true spatial distance.

Conversely, poor to moderate agreement was observed for granular metrics, including double support time (0.484), single support time (0.484), and swing time (0.489). Pelvic kinematics yielded poor agreement across the sagittal (0.547), coronal (0.302), and transverse (0.603) planes. The IMU failed to reliably capture any asymmetry metrics, yielding negative global ICC values for stride length and velocity asymmetry (ICC = −0.090 and −0.133, respectively; Table 3).

**Figure 3:**
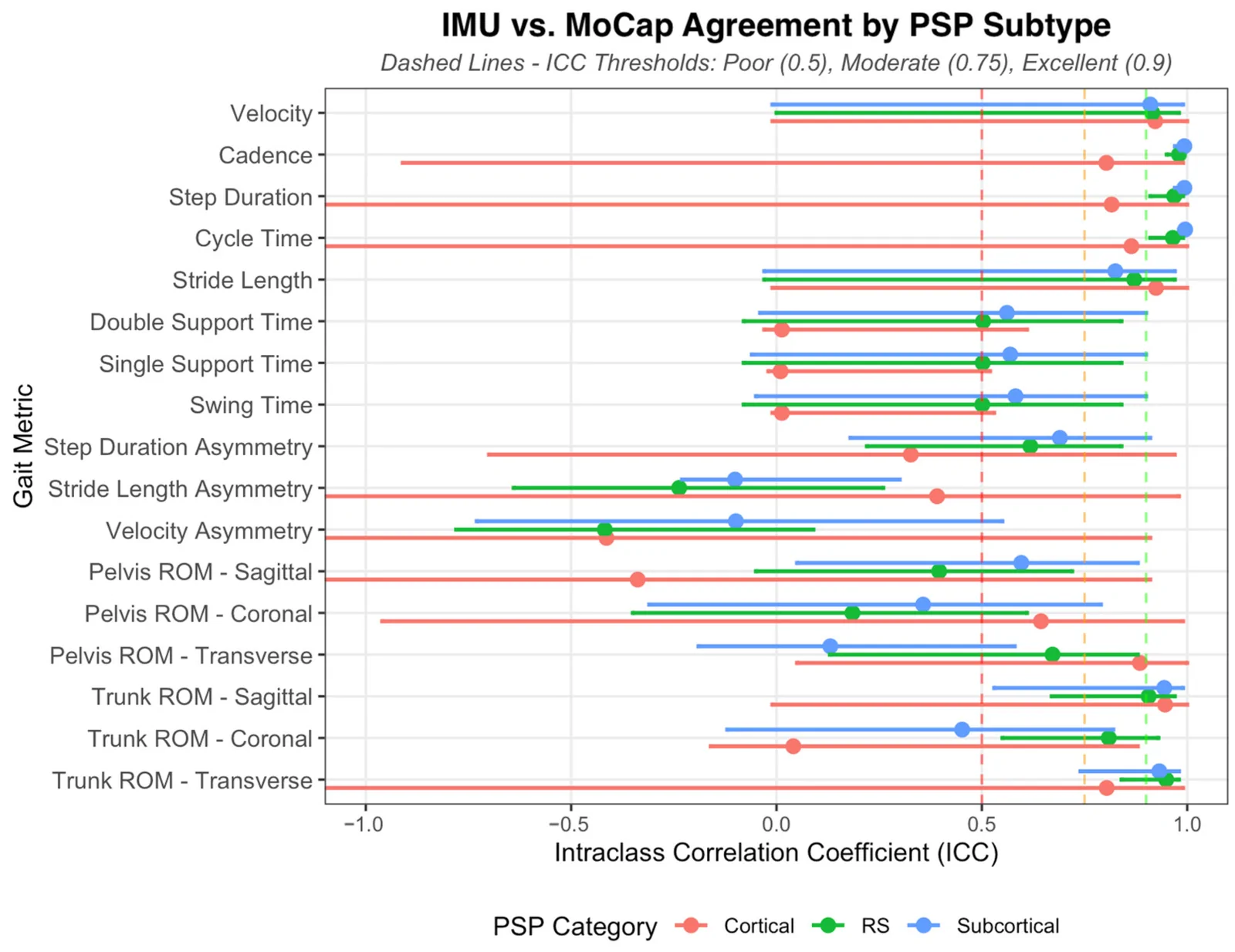
Stratified Intraclass Correlation Coefficients for all 17 metrics. Dashed lines indicate standard agreement thresholds (red = poor, yellow = moderate, green = excellent). Note: the cortical subtype group contained *n* = 3 participants, yielding large ranges for recorded metrics.

We conducted exploratory analyses stratified by PSP phenotype. The RS and subcortical subtypes generally maintained the agreement patterns established in the global analysis, with excellent agreement in macro-temporal metrics and trunk kinematics, but degraded performance in support phases. The cortical cohort only contained *n* = 3 participants, yielding large variability across all measurements and making interpretations difficult. Due to the small number of distinct PSP subtypes these descriptive results are entirely exploratory and will require replication is future larger cohort of participants. Stratified ICCs and 95% Confidence Intervals are visualized in Figure 3.

### 3.4. Association Between Clinical Severity and Measurement Error

To determine if pathological gait mechanics are associated with greater discrepancies in IMU measurements, we analyzed the relationship between absolute measurement error and clinical disease severity determined by PSPRS scores. Initial unadjusted Spearman rank correlations revealed significant positive relationships between PSPRS scores and absolute error in double support time (ρ = 0.41, *p* < 0.05), single support time (ρ = 0.43, *p* < 0.05), swing time (ρ = 0.43, *p* < 0.05), stride length asymmetry (ρ = 0.48, *p* < 0.05), sagittal pelvis ROM (ρ = 0.52, *p* < 0.05), and transverse trunk ROM (ρ = 0.38, *p* < 0.05; Table 4).

Multivariable linear regression was performed for all 17 metrics to isolate the effect of disease severity from confounding factors. Considering the small sample size relative to the number of covariates, these models are designated as exploratory and hypothesis-generating. Absolute error was modeled against PSPRS, while adjusting for mean MoCap gait velocity and patient age (Table 5). The multivariable models revealed that even when controlling for how slowly the patient walked and their age, a higher PSPRS score remained an independent, statistically significant predictor of increased measurement error for double support time (adj. *p* < 0.05), single support time (adj. *p* < 0.05), swing time (adj. *p* < 0.05), sagittal pelvis ROM (adj. *p* < 0.05), and transverse trunk ROM (adj. *p* < 0.05). Notably, while stride length asymmetry demonstrated a significant unadjusted correlation with PSPRS (*p* < 0.05), it lost significance in the adjusted multivariable model (adj. *p* = 0.236), indicating that the IMU’s failure to capture asymmetry is primarily explained by reduced walking velocity rather than the unique pathological features of severe PSP.

To determine if cognitive impairment drove measurement error or if it confounded motor severity, a second multivariate model was utilized that included the patients MoCA scores as an additional covariate to gait velocity and age. This model showed that the addition of MoCA scores did not present as a predictor of measurement error in any of the recorded metrics. It also did not alter any primary relationships, as PSPRS remained an independent predictor of measurement error for single and double support time, swing time, and sagittal pelvis ROM. However, PSPRS did lose significance as a predictor of transverse trunk ROM (*p* = 0.116).

### 3.5. Static Postural Sway Validation

**Figure 4:**
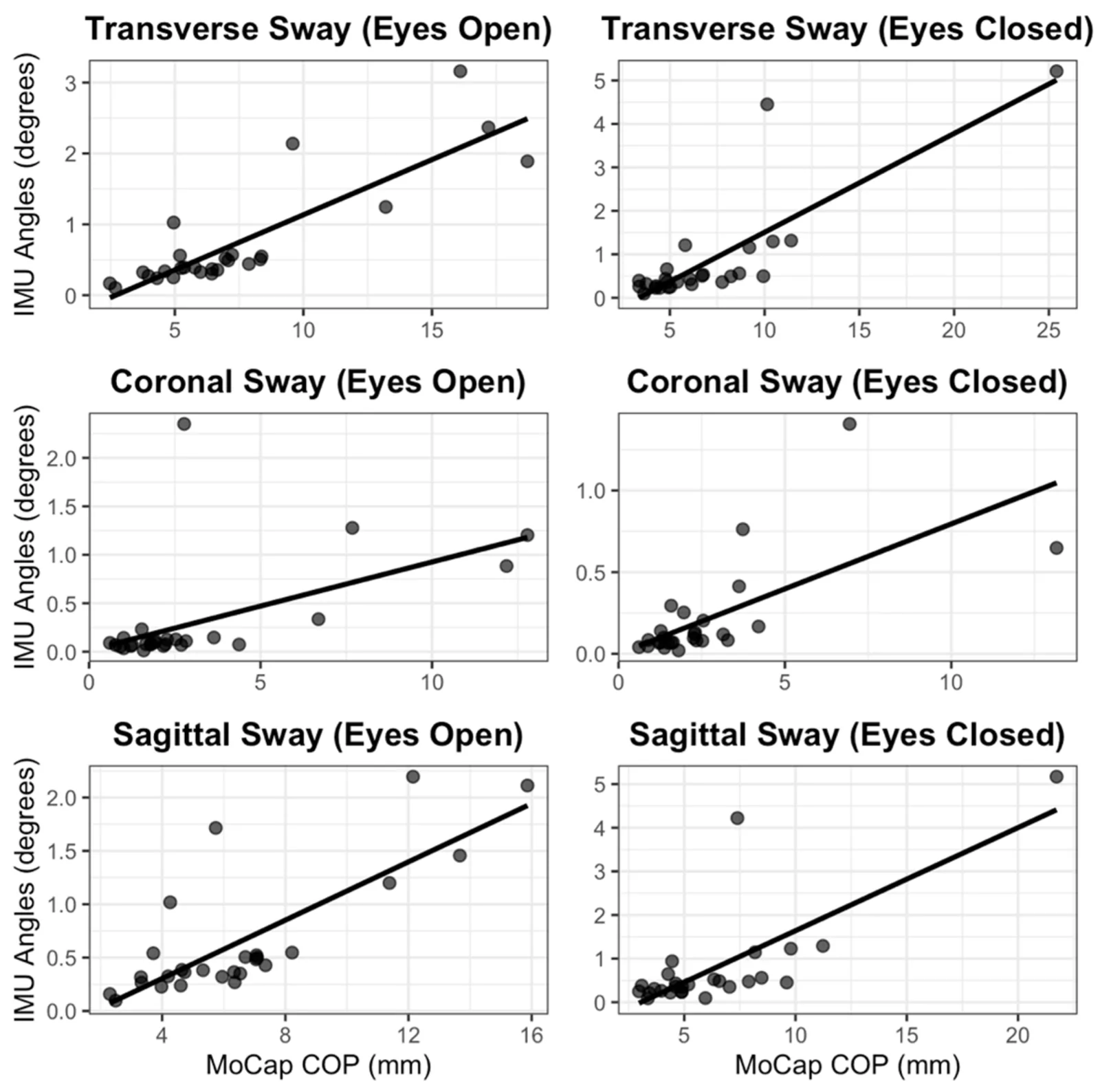
Correlations between sway variables captured by the MoCap and IMU systems. MoCap measurements are in mm, while IMU measurements are in degrees.

We utilized Spearman Rank Correlations to determine the relative association and rank-order correspondence of postural sway variables due to the difference between measurement units of both systems. The IMU demonstrated strong rank-order correspondence across all conditions and directions (Figure 4). Transverse sway showed strong positive correlations in the EO (ρ = 0.82, *p* < 0.05) and EC (ρ = 0.756, *p* < 0.05) conditions. Most notably with PSP, which characterizes backward disequilibrium and retrocollis, sagittal sway exhibited strong correlations in the EO (ρ = 0.719, *p* < 0.05) and EC (ρ = 0.662, *p* < 0.05) conditions. Coronal sway yielded similar significant relationships for both conditions (ρ = 0.612 and ρ = 0.662, respectively; *p* < 0.05).

While the IMU could capture the relative severity of postural instability, it failed to demonstrate absolute agreement with the MoCap system when calculating the Romberg Ratio in both conditions. ICC for the Romberg Ratio was poor between the two systems (ICC = 0.29, 95% CI [−0.09, 0.60], *p* = 0.067). This lack of agreement indicates that while both systems can detect an increase in instability, the angular kinematic changes measured by the IMU do not scale proportionally with the linear center of pressure displacement recorded by the MoCap system.

## 4. Discussion

In this study, we evaluated the concurrent validity and absolute agreement of a commercial six-sensor IMU system and its software output against a gold standard optical marker-based MoCap system for assessing gait, kinematic ROM, and postural sway in a cohort of participants with PSP. We test this common commercial IMU system and software as it is highly likely to be used in clinical settings where time and expertise for custom sensor set up and analyses are limited. We additionally sought to determine if the severity of PSP would independently alter the measurement accuracy of the IMU. Overall, our findings suggest that the commercial APDM Opal IMU system has excellent agreement for foundational temporal gait parameters and strong concurrent validity for measuring the severity of postural sway. However, the accuracy of this system degrades when analyzing precise gait phases and spatial asymmetries. Our multivariable modeling revealed that greater clinical disease severity as measured by the PSPRS independently predicts higher measurement error in specific kinematic and temporal metrics, indicating that the pathological movements associated with PSP are strongly associated with measurement discrepancies in the commercial IMU system algorithms. Given that gait and balance deficits are cardinal features of this disease, these results provide novel insights into an under-researched population, highlighting the importance of validating device performance when deploying digital endpoints in complex neurodegenerative populations.

The IMU demonstrated excellent agreement in capturing foundational temporal variables, yielding near perfect agreement for cadence, step duration, and cycle time. However, this temporal accuracy was accompanied by a significant underestimation of stride length, resulting in a negative bias that artificially reduced the calculated gait velocity. When interpreting these results, it is important to emphasize that high ICC values reflect strong rank-order consistency, rather than absolute interchangeability. As shown with our Bland–Altman and TOST analyses, high correlation can coexist with significant systematic bias, reinforcing that the two systems are not directly substitutable. While commercial IMU systems may be used in home or clinical settings to track mobility trends in patients with PSP [10,24], IMU-derived walking speed and stride length must be interpreted with caution. Existing literature evaluating IMU assessments in neurodegenerative diseases, such as Parkinson’s disease, generally reports more robust spatial tracking compared to our findings in PSP [10,25]. However, the severity of pathological motor features of PSP [5,7,26], likely affect the gait and balance parameters measured by commercial algorithms designed around normative inverted pendulum walking models, leading to the observed drift in measurements. Furthermore, negative ICC values, as seen with spatial and temporal asymmetry metrics, occur when the within-subject variance exceeds the between-subject variance, indicating a complete lack of agreement.

Agreement between the IMU and MoCap systems greatly diminished when examining granular aspects of the gait cycle, notably single and double support, swing times, and measurements of spatial and temporal asymmetry. In pathological gait, determining the exact moment of heel-strike and toe-off becomes challenging due to the characteristics of the disease [27,28]. Since patients with PSP typically exhibit shuffling steps [7], prolonged double-support phases [25,29], and poor foot clearance [30], the IMU algorithm struggles to identify the acceleration peaks typically used to segment these specific gait phases. This aligns with limitations observed in other severe neurological populations such as cerebellar ataxia [9], where ataxic foot placement confounds the gait event detection. As a result, current commercial IMU systems seem unable to measure these granular gait cycle variables as accurate digital biomarkers for disease progression.

Inclusion of the TOST procedure highlighted a critical distinction between agreement and equivalence in pathological populations. Despite demonstrating excellent ICCs for temporal related metrics, the IMU system failed to achieve statistical equivalence with the MoCap system for all spatiotemporal variables and pelvic kinematics. This failure in achieving equivalency may be influenced by the IMU algorithms introducing systematic offsets, as seen with measurements of stride length, that ultimately push the 90% confidence intervals of measurement error outside the defined equivalency bounds. Interestingly, sagittal and transverse trunk ROM were the only metrics to achieve equivalence. This suggests that while pathological gait mechanics disrupt the algorithms responsible for segmenting the gait cycle, the sensors remain generally robust for tracking upper-body kinematics. Ultimately, the TOST procedure suggests that the MoCap and IMU systems have systematic differences that researchers must account for when determining which hardware modality to employ during clinical studies.

Aside from assessing mean differences in recorded metrics, the width of the limits of agreement reported in Table 1 help signify whether the observed discrepancies are clinically acceptable. While metrics like cadence showed narrow agreement intervals (Bias: 0.24, LoA: [−3.63, 4.11] steps/min), other metrics such as stride length (Bias: 9.29, LoA: [2.52, 16.05] cm) and double support time (Bias: 8.65, LoA: [2.14, 15.16] %) demonstrated wide limits of agreement. These wide limits of agreement indicate that the individual measurement noise was high and therefore lack the precision for clinical diagnosis. However, whether these discrepancies compromise longitudinal tracking remains unknown, and dedicated studies tracking changes over time are needed to determine if these error bounds remain stable and if the devices used can reliably detect motor decline despite baseline measurement offsets.

An important finding in this study is that higher PSPRS scores were independently associated with greater between-system discrepancies for specific support phases and kinematics after adjusting for age and gait velocity. While the variance explained by the models ranged from 0.10 to 0.31 for significant predictors, the ability of the PSPRS scores to account for this amount of variance indicates a strong disease-related effect, with the remaining variance in measurement error likely being attributable to technological and physiological noise. This effect was also retained when the model accounted for cognitive impairment based on MoCA scores. While these scores did not independently predict measurement error for any metric, their inclusion in the model caused PSPRS to lose its predictive significance for transverse trunk ROM. This is likely due to collinearity, as axial rigidity and cognitive decline often present together in late-stage PSP. Together, these results suggest that the IMU’s algorithm disruption is driven by overall motor pathology rather than cognitive factors.

Since this study evaluated commercial software outputs rather than raw signals, these discrepancies do not reflect a failure of the IMU hardware itself. Rather, while raw IMU sensor accuracy generally remains robust, the observed errors demonstrate limitations arising from layers of data processing: sensor placement assumptions, automated gait event detection algorithms, proprietary software processing, and biomechanical model assumptions derived from normative inverted pendulum models. While the IMU hardware records acceleration and angular velocity, these software layers may fail to translate the signals accurately when exposed to atypical movement patterns seen with PSP. Therefore, caution is recommended in future longitudinal clinical studies, as increasing error rates tied to disease progression may mask or exaggerate clinical decline. Researchers must account for this disease-driven error when relying on wearable sensors and readily available commercial software for longitudinal studies.

Our evaluation of postural sway revealed statistically significant rank-order correspondence across all sensory conditions and directions, demonstrating that the IMU system can capture the cross-sectional severity of postural instability, but does not directly reproduce linear COP displacement. Since angular kinematic tilt and linear COP displacement represent different physical constructs, this correlation indicates association rather than interchangeability. This is particularly important for PSP, as the correlations in sagittal sway validate the IMU’s utility in monitoring backward disequilibrium that underlie sever fall risk [4,7,26]. To test whether these constructs could be normalized into a shared metric, we evaluated the Romberg Ratio. Although dimensionless ratios theoretically bypass unit differences, the two systems were unable to demonstrate absolute agreement (ICC = 0.29). Since the IMU measures angular tilt in degrees and the MoCap system measures linear center of pressure in millimeters, the scaling between the two systems becomes decoupled during sensory manipulation. This indicates that while IMU systems are useful tools for tracking the severity of sway over time, researchers will be unable to translate historical, MoCap system derived ratios directly onto IMU-derived data, and IMU variables must be analyzed strictly on their own normative scales.

This study comprised a few limitations. First, this validation study was conducted using a specific commercial IMU sensor system and its associated software. The system was selected based on common use in prior studies and published validation data in other neurological populations [11,31,32]. Since these devices utilize proprietary black-box algorithms, it is difficult to determine the exact thresholds at which the sensor will fail to identify pathological gait events, implying that these results may not universally translate to all wearable devices currently on the market. However, this allowed us to replicate a common scenario where in a clinical setting, a commercially available software is much more likely to be used to obtain gait measurements rather than a custom IMU set up and analysis algorithm due to limited time and technical gait analysis expertise. Second, the gait data collection was performed on a 7 m instrumented walkway, which may not reflect the biomechanical complexities of regular daily living. When in a home or in public, turns, obstacles and cognitive dual tasking will introduce additional variability not accounted for in this study. Third, while a sample size of 30 participants is robust for a pilot study evaluating a rare disease, the assessment of device performance across heterogenous phenotypes in PSP is limited. Consequently, the multivariable regression models should be strictly interpreted as exploratory, providing preliminary evidence of the association between clinical severity and measurement error. Finally, given the sample size and evaluation of 17 related biomechanical outputs, multiple statistical testing presents an inherent risk of false-positive findings. While applying error corrections was deliberately avoided to prevent type II errors, these findings should be viewed as hypothesis-generating observations that require replication in large-scale trials.

Future studies should focus on closing the gap between clinical accessibility and algorithmic accuracy for neurodegenerative populations. There is a critical need for the development of disease-specific, open-source algorithms trained on atypical movement data rather than normative models. By developing algorithms trained to identify acceleration profiles associated with impaired gait patterns, the accuracy of granular gait cycle measurements may be drastically improved. Furthermore, longitudinal studies in heterogenous PSP subtypes are required to determine how IMU measurement error evolves over time within the same patient as the disease progresses. Validating these systems in unconstrained environments with continuous monitoring is essential to establish robust, digital endpoints for future clinical trials.

## 5. Conclusions

In summary, this study demonstrates that commercial IMU systems provide a highly accessible, clinical alternative to MoCap systems for capturing foundational gait parameters and tracking relative postural instability in PSP. However, equivalence testing revealed that IMU and MoCap systems are not directly interchangeable, as systematic biases prevent equivalence for most gait metrics, while only trunk kinematics attained equivalence. Crucially, multivariable modeling demonstrated that between-system discrepancies are independently associated with the motor severity of PSP rather than cognitive decline. Clinicians and researchers must recognize that while commercial IMU systems are useful tools for longitudinal mobility tracking, the out-of-the-box proprietary algorithms associated with these devices are not yet equipped to accurately capture atypical movement patterns typically associated with PSP. Ultimately, the inability of the IMU system tested here to attain equivalence with the MoCap system for most metrics underscores the importance of careful consideration when selecting hardware for clinical studies. Researchers should adhere to a single hardware modality, especially in longitudinal studies, as mixing data sources may directly confound the measurement of progressive motor decline.

## Figures and Tables

**Table 1:** Bland–Altman results for all 17 metrics. Reported values are the means and standard deviations of both collection methods, the Bland–Altman bias and limits of agreement (LoA), the Wilcoxon Signed Rank *p*, and the TOST *p*.

Metric	MoCap	IMU	Bias	LoA	*p*	TOST *p*
Velocity (cm/s)	79.48 ± 19.23	71.99 ± 18.63	7.49	[1.40, 13.59]	<0.05	1
Cadence (steps/min)	100.76 ± 11.42	100.52 ± 11.59	0.24	[−3.63, 4.11]	0.382	0.508
Step Duration (s)	0.61 ± 0.07	0.61 ± 0.08	0.00	[−0.03, 0.03]	0.543	0.936
Cycle Time (s)	1.21 ± 0.15	1.22 ± 0.15	0.00	[−0.06, 0.06]	0.871	0.686
Stride Length (cm)	94.14 ± 17.58	84.85 ± 17.16	9.29	[2.52, 16.05]	<0.05	0.778
Double Support Time (% gait cycle)	34.22 ± 6.68	25.58 ± 6.81	8.65	[2.14, 15.16]	<0.05	1
Single Support Time (% gait cycle)	33.01 ± 3.32	37.25 ± 3.42	−4.23	[−7.68, −0.79]	<0.05	1
Swing Time (% gait cycle)	33.01 ± 3.31	37.18 ± 3.40	−4.17	[−7.59, −0.75]	<0.05	1
Step Duration Asymmetry (% diff.)	0.63 ± 8.89	0.25 ± 10.08	0.38	[−15.68, 16.43]	0.289	0.803
Stride Length Asymmetry (% diff.)	0.44 ± 3.14	−1.82 ± 3.56	2.26	[−7.52, 12.05]	<0.05	0.773
Velocity Asymmetry (% diff.)	−0.14 ± 8.23	−1.26 ± 3.43	1.12	[−17.45, 19.70]	0.746	0.521
Pelvis ROM—Sagittal (deg)	5.99 ± 2.17	5.23 ± 1.74	0.75	[−2.79, 4.29]	<0.05	0.156
Pelvis ROM—Coronal (deg)	4.89 ± 1.71	4.70 ± 2.12	0.19	[−4.28, 4.66]	0.289	0.652
Pelvis ROM—Transverse (deg)	7.96 ± 2.84	9.73 ± 2.51	−1.76	[−5.64, 2.11]	<0.05	0.826
Trunk ROM—Sagittal (deg)	4.11 ± 1.72	4.50 ± 1.64	−0.39	[−1.48, 0.70]	<0.05	<0.05
Trunk ROM—Coronal (deg)	4.00 ± 1.10	4.03 ± 1.15	−0.03	[−2.11, 2.05]	0.529	0.876
Trunk ROM—Transverse (deg)	9.15 ± 3.24	8.66 ± 3.04	0.49	[−1.32, 2.30]	<0.05	<0.05

**Table 2:** Mean Absolute Error and Root Mean Square Error for all 17 metrics.

Metric	MAE	RMSE
Velocity	7.59	8.091
Cadence	1.403	1.956
Step Duration	0.010	0.015
Cycle Time	0.020	0.030
Stride Length	9.289	9.889
Double Support Time	8.670	9.243
Single Support Time	4.286	4.573
Swing Time	4.234	4.510
Step Duration Asymmetry	5.896	8.062
Stride Length Asymmetry	4.293	5.404
Velocity Asymmetry	7.115	9.386
Pelvis ROM—Sagittal	1.483	1.927
Pelvis ROM—Coronal	1.644	2.252
Pelvis ROM—Transverse	2.050	2.625
Trunk ROM—Sagittal	0.522	0.673
Trunk ROM—Coronal	0.716	1.042
Trunk ROM—Transverse	0.784	1.03

**Table 3:** Intraclass Correlation Coefficients for all 17 metrics across the cohort of participants.

Metric	Estimate	95% CI	*p*
Velocity	0.915	[−0.01, 0.98]	<0.05
Cadence	0.986	[0.97, 0.99]	<0.05
Step Duration	0.981	[0.96, 0.99]	<0.05
Cycle Time	0.980	[0.96, 0.99]	<0.05
Stride Length	0.858	[−0.03, 0.97]	<0.05
Double Support Time	0.484	[−0.06, 0.82]	<0.05
Single Support Time	0.484	[−0.07, 0.82]	<0.05
Swing Time	0.489	[−0.07, 0.82]	<0.05
Step Duration Asymmetry	0.636	[0.36, 0.81]	<0.05
Stride Length Asymmetry	−0.090	[−0.37, 0.24]	0.716
Velocity Asymmetry	−0.133	[−0.48, 0.24]	0.757
Pelvis ROM—Sagittal	0.547	[0.24, 0.76]	<0.05
Pelvis ROM—Coronal	0.302	[−0.07, 0.60]	0.053
Pelvis ROM—Transverse	0.603	[0.08, 0.83]	<0.05
Trunk ROM—Sagittal	0.922	[0.73, 0.97]	<0.05
Trunk ROM—Coronal	0.564	[0.26, 0.77]	<0.05
Trunk ROM—Transverse	0.947	[0.86, 0.98]	<0.05

**Table 4:** Spearman rank correlations between absolute error of MoCap and IMU devices and PSPRS scores across all 17 metrics.

Metric	ρ	*p*
Velocity	−0.14	0.452
Cadence	0.30	0.108
Step Duration	0.22	0.241
Cycle Time	0.28	0.132
Stride Length	−0.23	0.225
Double Support Time	0.41	<0.05
Single Support Time	0.43	<0.05
Swing Time	0.43	<0.05
Step Duration Asymmetry	0.26	0.159
Stride Length Asymmetry	0.48	<0.05
Velocity Asymmetry	0.23	0.229
Pelvis ROM—Sagittal	0.52	<0.05
Pelvis ROM—Coronal	−0.08	0.668
Pelvis ROM—Transverse	−0.03	0.865
Trunk ROM—Sagittal	−0.12	0.517
Trunk ROM—Coronal	0.21	0.270
Trunk ROM—Transverse	0.38	<0.05

**Table 5:** Multivariable linear regression for absolute error of metrics controlling for gait velocity and age (M1), as well as gait velocity, age, and MoCA score (M2). The reported adjusted *p*-values and R^2^ values reflect the independent predictive value of the PSPRS score within each respective model. PSPRS score predicted measurement error in double and single support times, swing time, and sagittal pelvic and transverse trunk ROM.

Metric	M1 Adj. *p*	M1 Adj. R^2^	M2 Adj. *p*	M2 Adj. R^2^
Velocity	0.983	−0.015	0.682	0.013
Cadence	0.385	0.077	0.692	0.125
Step Duration	0.560	0.263	0.999	0.339
Cycle Time	0.491	0.208	0.954	0.360
Stride Length	0.288	−0.057	0.199	−0.060
Double Support Time	<0.05	0.096	<0.05	0.132
Single Support Time	<0.05	0.120	<0.05	0.156
Swing Time	<0.05	0.139	<0.05	0.182
Step Duration Asymmetry	0.449	−0.070	0.722	−0.043
Stride Length Asymmetry	0.236	0.360	0.145	0.368
Velocity Asymmetry	0.374	−0.050	0.471	−0.085
Pelvis ROM—Sagittal	<0.05	0.227	<0.05	0.198
Pelvis ROM—Coronal	0.779	0.115	0.921	0.091
Pelvis ROM—Transverse	0.606	−0.098	0.606	−0.141
Trunk ROM—Sagittal	0.948	0.081	0.916	0.045
Trunk ROM—Coronal	0.836	−0.067	0.820	−0.109
Trunk ROM—Transverse	<0.05	0.312	0.116	0.487

## Data Availability

The data supporting the conclusions of this article will be made available by the authors on request.

## Supplementary Materials

The following supporting information can be downloaded at: https://www.mdpi.com/article/10.3390/s26165105/s1, Table S1: Expanded Bland-Altman results for all 17 metrics; Table S2: TOST procedure equivalence bounds derived from the computation of one half of the MoCap systems standard deviation, and the 90% confidence intervals for the mean differences of all metrics.
