# Supplementary material for: Concurrent Validity and Between-System Agreement of a Commercial Wearable Inertial Sensor System for Gait and Postural Sway Assessment in Progressive Supranuclear Palsy
Source: Sensors (Basel). 2026 Aug 12;26(16):5105. doi: 10.3390/s26165105 (PMC13517926; doi:10.3390/s26165105)
Supplement: Supplementary file 1 [file sensors-26-05105-s001.zip › Supplementary Analysis Code.pdf]

```
##---- Workspace Setup ----
# Load Libraries
library(dplyr)
library(tidyr)
library(blandr)
library(purrr)
library(psych)
library(ggplot2)
library(stringr)
library(TOSTER)
library(readr)
library(readxl)
library(patchwork)

# Set Working Directory and Load Data
setwd()
data <- read.csv(file = "", header = TRUE)
clin_data <- read.csv(file = "", header = TRUE)

##---- Data Wrangling ----
# Select "pspeep" in Patient ID and Remove NAs
psp_data <- data %>%
  filter(grepl("pspeep", X)) %>%
  drop_na()

# Select Clinical Values for Identified Patients
psp_clin_data <- clin_data %>%
  filter(.[[26]] %in% psp_data[[1]]) %>%
  arrange(match(.[[26]], psp_data[[1]]))

##---- Define Variable Pairs ----
# TOST Values are 0.5 * MoCap Std Dev
metrics <- list(
  c("Velocity_MoCap_mean", "Velocity_IMU_mean", "Velocity", 5),
  c("Cadence_MoCap_mean", "Cadence_IMU_mean", "Cadence", 5.71),
  c("Step.Duration_MoCap_mean", "Step.Duration_IMU_mean", "Step Duration", 0.04),
  c("Cycle.Time_MoCap_mean", "Cycle.Time_IMU_mean", "Cycle Time", 0.07),
  c("Stride.Length._MoCap_mean", "Stride.Length._IMU_mean", "Stride Length", 8.79),
  c("Double.Support.Time_MoCap_mean", "Double.Support.Time_IMU_mean", "Double Support Time", 3.34),
  c("Single.Support.Time_MoCap_mean", "Single.Support.Time_IMU_mean", "Single Support Time", 1.66),
  c("Swing.Time_MoCap_mean", "Swing.Time_IMU_mean", "Swing Time", 1.65),
  c("Step.Duration.Asymmetry_MoCap_mean", "Step.Duration.Asymmetry_IMU_mean", "Step Duration Asymmetry",
4.44),
  c("Stride.Length.Asymmetry_MoCap_mean", "Stride.Length.Asymmetry_IMU_mean", "Stride Length Asymmetry",
1.57),
  c("Velocity.Asymmetry._MoCap_mean", "Velocity.Asymmetry._IMU_mean", "Velocity Asymmetry", 4.12),
  c("Pelvis_ROM_X_MoCap_mean", "Pelvis_ROM_X_IMU_mean", "Pelvis ROM - Sagittal", 1.09),
  c("Pelvis_ROM_Y_MoCap_mean", "Pelvis_ROM_Y_IMU_mean", "Pelvis ROM - Coronal", 0.85),
  c("Pelvis_ROM_Z_MoCap_mean", "Pelvis_ROM_Z_IMU_mean", "Pelvis ROM - Transverse", 1.42),
  c("Trunk_ROM_X_MoCap_mean", "Trunk_ROM_X_IMU_mean", "Trunk ROM - Sagittal", 0.86),
  c("Trunk_ROM_Y_MoCap_mean", "Trunk_ROM_Y_IMU_mean", "Trunk ROM - Coronal", 0.55),
  c("Trunk_ROM_Z_MoCap_mean", "Trunk_ROM_Z_IMU_mean", "Trunk ROM - Transverse", 1.62)
)

# Dynamically Get Mean Difference Columns
diff_metrics <- map_chr(metrics, ~ sub("_MoCap_mean", "_Mean_Difference", .x[1]))

##---- Bland-Altman Function ----
gait_analysis <- function(vars, df, group_var = NULL) {
  mocap_var <- vars[1]
  imu_var <- vars[2]
  title_name <- vars[3]
  eq_bound <- as.numeric(vars[4])

  # Bland-Altman
  ba_stats <- blandr.statistics(df[[mocap_var]], df[[imu_var]])

  # Extract Means and Differences
```

```
  plot_df <- data.frame(
    plot_means = ba_stats$means,
    plot_diffs = ba_stats$differences,
    group_col = df[[group_var]]
  )

  # Plot Results
  x_max <- max(plot_df$plot_means, na.rm = TRUE)
  p <- ggplot(plot_df, aes(x = plot_means, y = plot_diffs)) +
    # Horizontal Lines for Bias and Limits of Agreement
    geom_hline(yintercept = ba_stats$bias, color = "black", linetype = "solid") +
    geom_hline(yintercept = ba_stats$upperLOA, color = "black", linetype = "dashed") +
    geom_hline(yintercept = ba_stats$lowerLOA, color = "black", linetype = "dashed") +
    annotate("text", x = x_max, y = ba_stats$bias, label = paste("Bias: ", round(ba_stats$bias, 2)), vjust =
-0.8, hjust = 1, size = 3.5, fontface = "bold")
  # Color Data Points by PSP Category
  p <- p + geom_point(aes(color = group_col), size = 2, alpha = 0.7) +
    labs(color = "PSP Category")
  # Edit Labels and Theme
  p <- p +
    labs(title = paste("Mean", title_name),
         x = "Means",
         y = "Differences") +
    theme_bw() +
    theme(legend.position = "bottom",
          plot.title = element_text(hjust = 0.5),
          panel.border = element_blank(),
          axis.line = element_line(color = "black"))
  # Print Plot
  suppressWarnings(print(p))

  # Wilcoxon Signed Rank
  w_test <- wilcox.test(df[[mocap_var]], df[[imu_var]], paired = TRUE)

  # TOST Test
  tost_res <- t_TOST(
    x = df[[mocap_var]],
    y = df[[imu_var]],
    paired = TRUE,
    eqb = eq_bound
  )

  # Calculate MAE and RMSE
  diff_vec <- df[[mocap_var]] - df[[imu_var]]
  mae <- mean(abs(diff_vec), na.rm = TRUE)
  rmse <- sqrt(mean(diff_vec^2, na.rm = TRUE))

  # Calculate 90% CI on Mean Differences
  diff_ci <- stats::t.test(df[[mocap_var]], df[[imu_var]], paired = TRUE, conf.level = 0.90)$conf.int

  # Format Results
  data.frame(
    Metric = title_name,
    MoCap = sprintf("%.2f ± %.2f", mean(ba_stats$method1), sd(ba_stats$method1)),
    IMU = sprintf("%.2f ± %.2f", mean(ba_stats$method2), sd(ba_stats$method2)),
    Bias = sprintf("%.2f", ba_stats$bias),
    BiasCI = sprintf("[%.2f, %.2f]", ba_stats$biasLowerCI, ba_stats$biasUpperCI),
    LoA = sprintf("[%.2f, %.2f]", ba_stats$lowerLOA, ba_stats$upperLOA),
    LowLoACI = sprintf("[%.2f, %.2f]", ba_stats$lowerLOA_lowerCI, ba_stats$lowerLOA_upperCI),
    UpLoACI = sprintf("[%.2f, %.2f]", ba_stats$upperLOA_lowerCI, ba_stats$upperLOA_upperCI),
    MAE = sprintf("%.3f", mae),
    RMSE = sprintf("%.3f", rmse),
    P_Value = format.pval(w_test$p.value, eps = 0.05, digits = 3),
    TOST_P_Value = format.pval(max(tost_res$TOST$p.value), eps = 0.05, digits = 3),
    TOST_EQB = sprintf("[%.2f, %.2f]", tost_res$eqb$low_eq[1], tost_res$eqb$high_eq[1]),
    Mean_Diff_90_CI = sprintf("[%.2f, %.2f]", diff_ci[1], diff_ci[2])
  )
}
```

```
# BA and Wilcoxon Function Call
gait_results <- map_dfr(metrics, ~gait_analysis(.x, psp_data, "PSP_Category"))


##---- Spearman Test with Clinical Scores ----
# Add Absolute Error to psp_data
for (m in metrics) {
  abs_col_name <- paste0(m[3], "_Abs_Error")
  psp_data[[abs_col_name]] <- abs(psp_data[[m[1]]] - psp_data[[m[2]]])
}
# Get Column Names
abs_err_cols <- paste0(sapply(metrics, '[[', 3), "_Abs_Error")

# Compute Spearman Rho on Absolute Error and Clinical Scores
clin_cor <- corr.test(
  x = psp_data[, abs_err_cols],
  y = psp_clin_data[,5:9],
  method = "spearman",
  use = "pairwise",
  adjust = "holm")

# Format Matrices
r_format <- matrix(sprintf("%.2f", clin_cor$r), nrow = nrow(clin_cor$r), dimnames = dimnames(clin_cor$r))
p_format <- matrix(format.pval(clin_cor$p, eps = 0.05, digits = 3), nrow = nrow(clin_cor$p), dimnames =
dimnames(clin_cor$p))

# Interleave Matrices and Convert to Dataframe
r_df <- setNames(as.data.frame(r_format), paste0(colnames(r_format), "_Rho"))
p_df <- setNames(as.data.frame(p_format), paste0(colnames(p_format), "_p"))
clin_results <- cbind(r_df, p_df)[, order(c(seq_along(r_df), seq_along(p_df)))]

# Show Bland-Altman and Spearman Tables
print(gait_results)
print(clin_results)


##---- ICC Function ----
icc_analysis <- function(vars, df) {
  mocap_var <- vars[1]
  imu_var <- vars[2]
  title_name <- vars[3]

  # Isolate Columns
  ratings <- df %>%
    select(all_of(c(mocap_var, imu_var)))

  # ICC Calculation (lmer = FALSE for ANOVA)
  icc_res <- suppressWarnings(psych::ICC(ratings, lmer = FALSE))

  # Extract ICC2 (2-Way Random, Single Measurement, Absolute Agreement)
  icc_val <- icc_res$results[icc_res$results$type == "ICC2", "ICC"]
  icc_lower <- icc_res$results[icc_res$results$type == "ICC2", "lower bound"]
  icc_upper <- icc_res$results[icc_res$results$type == "ICC2", "upper bound"]
  p_val <- icc_res$results[icc_res$results$type == "ICC2", "p"]

  # Format Results
  data.frame(
    Metric = title_name,
    ICC_Estimate = sprintf("%.3f", icc_val),
    CI_95 = sprintf("[%.2f, %.2f]", icc_lower, icc_upper),
    P_Value = format.pval(p_val, eps = 0.05, digits = 3)
  )
}

# Execute Global ICC (All Patients)
global_icc_results <- map_dfr(metrics, ~icc_analysis(.x, psp_data))
print(global_icc_results)
```

```r
# Execute Stratified ICC (By PSP Category)
stratified_icc_results <- psp_data %>%
  # Split Dataframe into List Based on PSP_Category
  split(.$PSP_Category) %>%
  # Call ICC Function over Each subgroup
  map_dfr(function(sub_df) {
    map_dfr(metrics, ~icc_analysis(.x, sub_df))
  }, .id = "PSP_Category")
print(stratified_icc_results)

##---- ICC Visualization ----
# Global - Clean and Parse the Data
global_plot_data <- global_icc_results %>%
  # Extract Bounds from CI_95 String
  separate(CI_95, into = c("CI_lower", "CI_upper"), sep = ",") %>%
  mutate(
    ICC_Estimate = as.numeric(ICC_Estimate),
    CI_lower = parse_number(CI_lower),
    CI_upper = parse_number(CI_upper),
    # Reverse Metrics Factor Order
    Metric = factor(Metric, levels = rev(unique(Metric)))
  )

# Global - Generate the Forest Plot
global_icc_plot <- ggplot(global_plot_data, aes(x = ICC_Estimate, y = Metric)) +
  # Draw Points and Error Bars
  geom_point(size = 3.5, color = "#0072B2") +
  geom_errorbarh(aes(xmin = CI_lower, xmax = CI_upper),
                 height = 0.2, linewidth = 1, color = "#0072B2") +
  # Add Vertical Lines for ICC Thresholds
  geom_vline(xintercept = 0.50, linetype = "dashed", color = "red", alpha = 0.6) +   # Poor
  geom_vline(xintercept = 0.75, linetype = "dashed", color = "orange", alpha = 0.6) + # Moderate
  geom_vline(xintercept = 0.90, linetype = "dashed", color = "green", alpha = 0.6) +  # Excellent
  # Set X-axis Limits
  coord_cartesian(xlim = c(-1, 1)) +
  # Edit Labels and Theme
  labs(
    title = "IMU vs. MoCap Agreement",
    subtitle = "Dashed Lines - ICC Thresholds: Poor (0.5), Moderate (0.75), Excellent (0.9)",
    x = "Intraclass Correlation Coefficient (ICC)",
    y = "Gait Metric"
  ) +
  theme_bw() +
  theme(
    plot.title = element_text(hjust = 0.5, face = "bold", size = 14),
    plot.subtitle = element_text(hjust = 0.5, face = "italic", size = 10, color = "gray30"),
    panel.grid.minor = element_blank(),
    axis.text.y = element_text(size = 10)
  )
# Print Global Plot
print(global_icc_plot)


# Stratified - Clean and Parse Data
plot_data <- stratified_icc_results %>%
  mutate(
    ICC_Estimate = as.numeric(ICC_Estimate),
    # Extract Bounds from CI_95 String
    CI_lower = as.numeric(str_extract(CI_95, "(?<=\\[)[^,]+")),
    CI_upper = as.numeric(str_extract(CI_95, "(?<=,\\s)[^\\]]+")),
    # Reverse Metrics Factor Order
    Metric = factor(Metric, levels = rev(unique(Metric)))
  )

# Stratified - Generate Forest Plot
icc_plot <- ggplot(plot_data, aes(x = ICC_Estimate, y = Metric, color = PSP_Category)) +
  # Draw Points and Error Bars
```

```
  geom_point(position = position_dodge(width = 0.6), size = 2.5) +
  geom_errorbarh(aes(xmin = CI_lower, xmax = CI_upper),
                 position = position_dodge(width = 0.6), height = 0.3, linewidth = 0.8) +
  # Add Vertical Lines for ICC Thresholds
  geom_vline(xintercept = 0.50, linetype = "dashed", color = "red", alpha = 0.6) +   # Poor
  geom_vline(xintercept = 0.75, linetype = "dashed", color = "orange", alpha = 0.6) + # Moderate
  geom_vline(xintercept = 0.90, linetype = "dashed", color = "green", alpha = 0.6) +  # Excellent
  # Set X-axis Limits
  coord_cartesian(xlim = c(-1, 1)) +
  # Edit Labels and Theme
  labs(
    title = "IMU vs. MoCap Agreement by PSP Subtype",
    subtitle = "Dashed Lines - ICC Thresholds: Poor (0.5), Moderate (0.75), Excellent (0.9)",
    x = "Intraclass Correlation Coefficient (ICC)",
    y = "Gait Metric",
    color = "PSP Category"
  ) +
  theme_bw() +
  theme(
    plot.title = element_text(hjust = 0.5, face = "bold", size = 14),
    plot.subtitle = element_text(hjust = 0.5, face = "italic", size = 10, color = "gray30"),
    legend.position = "bottom",
    panel.grid.minor = element_blank(),
    axis.text.y = element_text(size = 10)
  )
# Print Stratified Plot
print(icc_plot)


##---- Multivariable Regression ----
# Get PSPRS Clinical Score and Append to psp_data
regression_data <- psp_data %>%
  mutate(Psprs = psp_clin_data$Psprs) %>%
  mutate(Moca = psp_clin_data$Moca)

# Function Definition - Four Tests for Each Metric
run_regression <- function(metric_name, df) {
  abs_err_col <- paste0(metric_name, "_Abs_Error")

  # Formula Definitions
  f_psprs <- as.formula(paste("`", abs_err_col, "` ~ Psprs", sep = ""))
  f_speed <- as.formula(paste("`", abs_err_col, "` ~ Velocity_MoCap_mean", sep = ""))
  f_age   <- as.formula(paste("`", abs_err_col, "` ~ Age_at_encounter", sep = ""))
  f_moca  <- as.formula(paste("`", abs_err_col, "` ~ Moca", sep = ""))
  f_multi <- as.formula(paste("`", abs_err_col, "` ~ Psprs + Velocity_MoCap_mean + Age_at_encounter", sep =
""))
  f_multi_moca <- as.formula(paste("`", abs_err_col, "` ~ Psprs + Velocity_MoCap_mean + Age_at_encounter +
Moca", sep = ""))

  # Run Models
  m_psprs <- lm(f_psprs, data = df)
  m_speed <- lm(f_speed, data = df)
  m_age   <- lm(f_age, data = df)
  m_moca  <- lm(f_moca, data = df)
  m_multi <- lm(f_multi, data = df)
  m_multi_moca <- lm(f_multi_moca, data = df)

  # Extract Single Psprs p-value and Multivariable Psprs p-value
  sum_psprs <- summary(m_psprs)
  sum_speed <- summary(m_speed)
  sum_age   <- summary(m_age)
  sum_moca  <- summary(m_moca)
  sum_multi <- summary(m_multi)
  sum_multi_moca <- summary(m_multi_moca)

  # Extract Coefficients if Model Successfully Ran
  p_psprs <- if("Psprs" %in% rownames(sum_psprs$coefficients)) sum_psprs$coefficients["Psprs", "Pr(>|t|)"]
else NA
```

```
  p_speed <- if("Velocity_MoCap_mean" %in% rownames(sum_speed$coefficients))
sum_speed$coefficients["Velocity_MoCap_mean", "Pr(>|t|)"] else NA
  p_age   <- if("Age_at_encounter" %in% rownames(sum_age$coefficients))
sum_age$coefficients["Age_at_encounter", "Pr(>|t|)"] else NA
  p_moca  <- if("Moca" %in% rownames(sum_moca$coefficients)) sum_moca$coefficients["Moca", "Pr(>|t|)"] else
NA
  p_multi <- if("Psprs" %in% rownames(sum_multi$coefficients)) sum_multi$coefficients["Psprs", "Pr(>|t|)"]
else NA
  p_multi_moca <- if("Psprs" %in% rownames(sum_multi_moca$coefficients)) sum_multi_moca$coefficients["Psprs",
"Pr(>|t|)"] else NA

  # Format Results
  data.frame(
    Metric = metric_name,
    PSPRS_P = format.pval(p_psprs, eps = 0.05, digits = 3),
    PSPRS_R2 = sprintf("%.3f", sum_psprs$adj.r.squared),
    Speed_P = format.pval(p_speed, eps = 0.05, digits = 3),
    Speed_R2 = sprintf("%.3f", sum_speed$adj.r.squared),
    Age_P = format.pval(p_age, eps = 0.05, digits = 3),
    Age_R2 = sprintf("%.3f", sum_age$adj.r.squared),
    Moca_P = format.pval(p_moca, eps = 0.05, digits = 3),
    Moca_R2 = sprintf("%.3f", sum_moca$adj.r.squared),
    Multi_P = format.pval(p_multi, eps = 0.05, digits = 3),
    Multi_R2 = sprintf("%.3f", sum_multi$adj.r.squared),
    Multi_Moca_P = format.pval(p_multi_moca, eps = 0.05, digits = 3),
    Multi_Moca_R2 = sprintf("%.3f", sum_multi_moca$adj.r.squared)
  )
}

# Execution
metric_names <- sapply(metrics, `[[`, 3)
regression_results <- map_dfr(metric_names, ~run_regression(.x, regression_data))
print(regression_results)


##---- Combined Plots ----
# 2x3 Bland-Altman Grid (With Row Tags)
# Define Metrics for Bland-Altman Plot
grid_metrics <- list(
  c("Cadence_MoCap_mean", "Cadence_IMU_mean", "Cadence"),
  c("Cycle.Time_MoCap_mean", "Cycle.Time_IMU_mean", "Cycle Time"),
  c("Step.Duration_MoCap_mean", "Step.Duration_IMU_mean", "Step Duration"),
  c("Double.Support.Time_MoCap_mean", "Double.Support.Time_IMU_mean", "Double Support Time"),
  c("Single.Support.Time_MoCap_mean", "Single.Support.Time_IMU_mean", "Single Support Time"),
  c("Swing.Time_MoCap_mean", "Swing.Time_IMU_mean", "Swing Time")
)

# Plot as Grid
get_grid_plot <- function(vars, df, index, group_var = "PSP_Category") {
  mocap_var <- vars[1]
  imu_var <- vars[2]
  title_name <- vars[3]

  # Calculate Bland-Altman Statistics
  ba_stats <- blandr.statistics(df[[mocap_var]], df[[imu_var]])

  # Extract Means and Differences
  plot_df <- data.frame(
    plot_means = ba_stats$means,
    plot_diffs = ba_stats$differences,
    group_col = df[[group_var]]
  )
  x_max <- max(plot_df$plot_means, na.rm = TRUE)

  # Dynamic A/B Tag
  row_tag <- if(index == 1) "A" else if(index == 4) "B" else NULL

  # Build Plot
```

```
  p <- ggplot(plot_df, aes(x = plot_means, y = plot_diffs)) +
    geom_hline(yintercept = ba_stats$bias, color = "black", linetype = "solid", linewidth = 0.8) +
    geom_hline(yintercept = ba_stats$upperLOA, color = "black", linetype = "dashed") +
    geom_hline(yintercept = ba_stats$lowerLOA, color = "black", linetype = "dashed") +
    geom_point(aes(color = group_col), size = 2.5, alpha = 0.7) +
    annotate("text", x = x_max, y = ba_stats$bias, label = paste("Mean: ", round(ba_stats$bias, 2)), vjust =
-0.8, hjust = 1, size = 3.5, fontface = "bold") +
    annotate("text", x = x_max, y = ba_stats$upperLOA, label = paste("ULA: ", round(ba_stats$upperLOA, 2)),
vjust = 1.5, hjust = 1, size = 3.5, fontface = "bold") +
    annotate("text", x = x_max, y = ba_stats$lowerLOA, label = paste("LLA: ", round(ba_stats$lowerLOA, 2)),
vjust = -0.8, hjust = 1, size = 3.5, fontface = "bold") +

    # Add A/B Tag
    labs(title = title_name, x = "Mean", y = "Difference", color = "PSP Category", tag = row_tag) +
    theme_bw() +
    theme(
      plot.title = element_text(hjust = 0.5, face = "bold", size = 12),
      panel.border = element_rect(color = "black", fill = NA),
      aspect.ratio = 1,
      # Style A/B Tag
      plot.tag = element_text(size = 18, face = "bold"),
      plot.tag.position = "topleft"
    )

  return(p)
}

# Generate Plots
plot_list <- imap(grid_metrics, ~get_grid_plot(.x, psp_data, .y))

# Arrange Plots
combined_grid <- wrap_plots(plot_list, ncol = 3, nrow = 2) +
  plot_layout(guides = "collect", axes = "collect") &
  theme(legend.position = "bottom")

# Display Grid
suppressWarnings(print(combined_grid))


##---- Sway Variables ####
sway_data <- read.csv(file = "", header = TRUE)
# Only Use Previously Identified Patients
psp_sway_data <- sway_data %>%
  filter(.[[1]] %in% psp_data[[1]]) %>%
  arrange(match(.[[1]], psp_data[[1]]))

# Define Pairs List
sway_pairs <- list(
  # Global Sway
  c("COPzero_RMS_EOxy", "Postural.Sway...Angles...RMS.Sway..degrees._EO", "Transverse Sway (Eyes Open)"),
  c("COPzero_RMS_ECxy", "Postural.Sway...Angles...RMS.Sway..degrees._EC", "Transverse Sway (Eyes Closed)"),
  # Mediolateral / Anteroposterior Sway
  c("COPzero_RMS_EOx", "Postural.Sway...Angles...RMS.Sway..Coronal...degrees._EO", "Coronal Sway (Eyes
Open)"),
  c("COPzero_RMS_ECx", "Postural.Sway...Angles...RMS.Sway..Coronal...degrees._EC", "Coronal Sway (Eyes
Closed)"),
  c("COPzero_RMS_EOy", "Postural.Sway...Angles...RMS.Sway..Sagittal...degrees._EO", "Sagittal Sway (Eyes
Open)"),
  c("COPzero_RMS_ECy", "Postural.Sway...Angles...RMS.Sway..Sagittal...degrees._EC", "Sagittal Sway (Eyes
Closed)")
)

# Sway Correlation (No BA Since Different Units)
sway_correlation <- function(mocap_var, imu_var, plot_title, df, plot_index) {
  sway_corr <- cor.test(
    x = df[[mocap_var]],
    y = df[[imu_var]],
    method = "spearman",
```

```
    exact = FALSE)

  # Display Tables
  cat("\n----", plot_title, "----\n")
  cat("Rho: ", round(sway_corr$estimate, 3), "\n")
  cat("p: ", format.pval(sway_corr$p.value, digits = 3), "\n")

  # Labeling Logic
  x_label <- if(plot_index %in% c(5,6)) "MoCap COP (mm)" else NULL
  y_label <- if(plot_index %in% c(1,3,5)) "IMU Angles (degrees)" else NULL

  # Build Plots
  p <- ggplot(df, aes(x = .data[[mocap_var]], y = .data[[imu_var]])) +
    geom_point(size = 2, alpha = 0.7) +
    geom_smooth(method = "lm", color = "black", se = FALSE) +
    labs(
      title = plot_title,
      x = x_label,
      y = y_label
    ) +
    theme_bw() +
    theme(plot.title = element_text(hjust = 0.5, face = "bold"))

  return(p)
}

# Run Over All Pairs
sway_plots <- purrr::imap(
  sway_pairs,
  ~sway_correlation(mocap_var = .x[1], imu_var = .x[2], plot_title = .x[3], df = psp_sway_data, plot_index =
.y)
)
# Display Plots in Grid
sway_grid <- wrap_plots(sway_plots, ncol = 2, nrow = 3) + plot_layout(guides = "collect")
suppressWarnings(print(sway_grid))


# Romberg Ratios
# Create New Dataframe
romberg_data <- data.frame(
  mocap_romberg = psp_sway_data$COP_RombergRatio,
  imu_romberg = psp_sway_data$Postural.Sway...Angles...RMS.Sway..degrees._EC /
psp_sway_data$Postural.Sway...Angles...RMS.Sway..degrees._EO
)

# Remove NA's (One Patient)
romberg_data <- na.omit(romberg_data)

# Perform ICC
icc_sway <- psych::ICC(romberg_data[, c("mocap_romberg", "imu_romberg")])
icc_sway_val <- icc_sway$results[icc_sway$results$type == "ICC2", "ICC"]
icc_sway_lb <- icc_sway$results[icc_sway$results$type == "ICC2", "lower bound"]
icc_sway_ub <- icc_sway$results[icc_sway$results$type == "ICC2", "upper bound"]
icc_sway_p <- icc_sway$results[icc_sway$results$type == "ICC2", "p"]
cat("\n--- Romberg Ratio ICC (Absolute Agreement) ---\n")
cat("ICC Estimate:", round(icc_sway_val, 3), "\n")
cat("95% CI: [", round(icc_sway_lb, 3), ",", round(icc_sway_ub, 3), "]\n")
cat("p-value:", format.pval(icc_sway_p, digits = 3), "\n")

# Bland-Altman Statistics
ba_romberg <- blandr.statistics(romberg_data$mocap_romberg, romberg_data$imu_romberg)

# Create the Bland-Altman Plot for the Romberg Ratio
p_ba_romberg <- ggplot(
  data.frame(Means = ba_romberg$means, Diffs = ba_romberg$differences),
  aes(x = Means, y = Diffs)
) +
  geom_hline(yintercept = ba_romberg$bias, color = "black", linetype = "solid", linewidth = 0.8) +
```

```
  geom_hline(yintercept = ba_romberg$upperLOA, color = "black", linetype = "dashed") +
  geom_hline(yintercept = ba_romberg$lowerLOA, color = "black", linetype = "dashed") +
  geom_point(size = 3, alpha = 0.7) +
  labs(
    title = "Romberg Ratio",
    x = "Mean",
    y = "Difference"
  ) +
  theme_bw() +
  theme(plot.title = element_text(hjust = 0.5, face = "bold"), aspect.ratio = 1)

suppressWarnings(print(p_ba_romberg))
```
