# Supplementary material for: Concurrent Validity and Between-System Agreement of a Commercial Wearable Inertial Sensor System for Gait and Postural Sway Assessment in Progressive Supranuclear Palsy
Source: Sensors (Basel). 2026 Aug 12;26(16):5105. doi: 10.3390/s26165105 (PMC13517926; doi:10.3390/s26165105)
Supplement: Supplementary file 1 [file sensors-26-05105-s001.zip › Supplementary Tables.pdf]

Supplemental Table S1. Expanded Bland-Altman results for all 17 metrics. Bias CI is the 95% confidence interval for the Bias value. Lower LoA CI is the 95% confidence interval for the lower Limits of Agreement value. Upper LoA CI is the 95% confidence interval for the upper Limits of Agreement value.

Metric	Bias	Bias CI	LoA	Lower LoA CI	Upper LoA CI
Velocity	7.49	[6.33, 8.65]	[1.40, 13.59]	[-0.61, 3.40]	[11.58, 15.59]
Cadence	0.24	[-0.50, 0.98]	[-3.63, 4.11]	[-4.90, -2.35]	[2.84, 5.39]
Step Duration	0	[-0.01, 0.01]	[-0.03, 0.03]	[-0.04, -0.02]	[0.02, 0.04]
Cycle Time	0	[-0.01, 0.01]	[-0.06, 0.06]	[-0.08, -0.04]	[0.04, 0.08]
Stride Length	9.29	[8.00, 10.58]	[2.52, 16.05]	[0.30, 4.75]	[13.83, 18.28]
Double Support Time	8.65	[7.41, 9.89]	[2.14, 15.16]	[-0.01, 4.28]	[13.01, 17.30]
Single Support Time	-4.23	[-4.89, -3.58]	[-7.68, -0.79]	[-8.81, -6.54]	[-1.93, 0.34]
Swing Time	-4.17	[-4.82, -3.52]	[-7.59, -0.75]	[-8.72, -6.46]	[-1.88, 0.37]
Step Duration Asymmetry	0.38	[-2.68, 3.43]	[-15.68, 16.43]	[-20.96, -10.39]	[11.14, 21.72]
Stride Length Asymmetry	2.26	[0.40, 4.13]	[-7.52, 12.05]	[-10.74, -4.30]	[8.82, 15.27]
Velocity Asymmetry	1.12	[-2.42, 4.66]	[-17.45, 19.70]	[-23.57, -11.34]	[13.58, 25.82]
Pelvis ROM - Sagittal	0.75	[0.08, 1.42]	[-2.79, 4.29]	[-3.95, -1.62]	[3.12, 5.45]
Pelvis ROM - Coronal	0.19	[-0.66, 1.04]	[-4.28, 4.66]	[-5.76, -2.81]	[3.19, 6.14]
Pelvis ROM - Transverse	-1.76	[-2.50, -1.03]	[-5.64, 2.11]	[-6.91, -4.36]	[0.83, 3.38]
Trunk ROM - Sagittal	-0.39	[-0.60, -0.19]	[-1.48, 0.70]	[-1.84, -1.12]	[0.34, 1.05]
Trunk ROM - Coronal	-0.03	[-0.43, 0.37]	[-2.11, 2.05]	[-2.79, -1.42]	[1.36, 2.73]
Trunk ROM - Transverse	0.49	[0.15, 0.83]	[-1.32, 2.30]	[-1.91, -0.72]	[1.70, 2.89]

Supplemental Table S2. TOST procedure equivalence bounds derived from the computation of one half of the MoCap systems standard deviation, and the 90% confidence intervals for the mean differences of all metrics.

Metric	TOST Eq. Bound	Mean Diff. 90% CI
Velocity	[-5.00, 5.00]	[6.53, 8.46]
Cadence	[-5.71, 5.71]	[-0.37, 0.85]
Step Duration	[-0.04, 0.04]	[-0.00, 0.00]
Cycle Time	[-0.07, 0.07]	[-0.01, 0.01]
Stride Length	[-8.79, 8.79]	[8.22, 10.36]
Double Support Time	[-3.34, 3.34]	[7.62, 9.68]
Single Support Time	[-1.66, 1.66]	[-4.78, -3.69]
Swing Time	[-1.65, 1.65]	[-4.71, -3.63]
Step Duration Asymmetry	[-4.44, 4.44]	[-2.16, 2.92]
Stride Length Asymmetry	[-1.57, 1.57]	[0.71, 3.81]
Velocity Asymmetry	[-4.12, 4.12]	[-1.82, 4.06]
Pelvis ROM - Sagittal	[-1.09, 1.09]	[0.19, 1.31]
Pelvis ROM - Coronal	[-0.85, 0.85]	[-0.52, 0.90]
Pelvis ROM - Transverse	[-1.42, 1.42]	[-2.38, -1.15]
Trunk ROM - Sagittal	[-0.86, 0.86]	[-0.57, -0.22]
Trunk ROM - Coronal	[-0.55, 0.55]	[-0.36, 0.30]
Trunk ROM - Transverse	[-1.62, 1.62]	[0.20, 0.78]
